# Response and recovery measures for two floods in north China during the nineteenth century: a comparative study

**DOI:** 10.1186/s40064-016-3642-y

**Published:** 2016-11-16

**Authors:** Yu Ye, Xiuqi Fang, Fan Li

**Affiliations:** 1 School of Geography, Beijing Normal University, 19 Xinjiekou Wai Street, Haidian District, Beijing, 100875 China; 2 Key Laboratory of Environment Change and Natural Disaster, Ministry of Education, BNU, Beijing, 100875 China

**Keywords:** Floods, The Yongding River, The Yellow River, Coping strategies, Impact of climate change

## Abstract

**Background:**

The process of human response to natural disasters and its mechanisms as revealed by historical events still has a broad significance for modern society. This study analyzed the disaster relief process and the social response for two floods in China: the Yongding River flood in 1801 and the Yellow River flood in 1841. These two floods reflect the different response processes between the national and provincial capitals during a stage of climate cooling and social transition in the Qing dynasty.

**Results:**

Applying methods of historical documents analysis and qualitatively comparative analysis to the materials such as *Relief Chronicles Authorized by the Emperor in XinYou* and *Flood Description in Bian Liang*, it shows that: (1) In 1801, the central government took on a lead position, from flood surveying to relief processes. However, local government and gentries played an important role in 1841. (2) In 1801, the government successfully undertook a series of relief measures addressing production, housing, food prices, taxes, and water conservancy and administration. In 1841, the response measures were relatively simple, focusing mainly on providing shelter and food for victims. (3) The government carried out long-term disaster prevention measures such as dredging channels after the flood in 1801. In 1841, however, the efforts were focused mainly on emergency rescue. (4) Refugees in the 1801 flood were effectively managed by a centralized authority. In 1841, regulation of the flooding was delayed by corruption and conflicts between officers, leading to an expansion of the disaster’s impact.

**Conclusions:**

Above results have led to the conclusion that disaster relief systems and response measures had a significant effect on the consequences of those floods. Various flood relief measures and natural disasters management regimes have implications for contemporary flood hazard mitigation.

## Background

The process of human response to natural disasters and its mechanisms as revealed by historical events still has a broad significance for the great challenge of modern society in coping with global climate change. The fifth report of the International Panel of Climate Change (IPCC) shows that global warming has resulted in more frequent extreme climatic events that have effects on agriculture and forest management, human health, and economic and social development (IPCC 2014). Continued global warming would most likely bring severe challenges for regional social and economic development and adaptation ability. Strengthening the research on the impact of extreme disasters on society and human coping strategies is not only an important scientific problem but also urgently needed for the healthy, stable and sustainable development of present and future societies in China.

In recent years, international studies examined the possible impact of abrupt climate change or natural disasters on social development. These studies have proven that the focus should be on both the feedback of the sub-system of society on abrupt climate change and the dynamics of climate change impact on society. For example, the abrupt climatic change that occurred in 2200 B.C. induced considerable land-use degradation and evidently caused the collapse of the Akkadian empire (Weiss et al. 1993). Cooling from A.D. 1560 to 1660 caused successive agro-ecological, socioeconomic, and demographic catastrophes, leading to the General Crisis of the seventeenth century in Europe (David et al. 2011). Widespread episodes of extreme drought due to as much as a 40% reduction in annual precipitation shaped the demise of the Maya civilization (Medina-Elizalde and Rohling 2012). Richard and Wagner (2010) analyzed the relationship between climate change and violent conflict in Europe over the last millennium. These above-mentioned international studies have revealed the relationships between historical extreme disasters and the decline of society, and the evolution of human civilization. However, it is necessary to reveal the process and dynamics of past climate change impacts, especially with regard to the impacts of climatic disasters.

The impact of historical, extreme climatic events and human adaptation has become one of the most important and most frequently researched areas in China, with abundant and extensive historical documents. Currently, this research can be classified into three types. First, case studies on the impact of natural disasters on important historical events; for example, the North Wei Dynasty moved the capital of Pingcheng (present-day Datong in Shanxi Province) to Luoyang in Henan Province during the second cold event of the North and South Dynasty of Weijin, 450–530s (Man et al. 2000). Second, estimates of the impact of climate change that compare historical climate or flood/drought series with a social and economic proxy index series; for example, population (Li 1999), food production (Hao et al. 2003), the scale of agricultural development (Zhang et al. 2005), the movement of the agricultural boundaries and grazing zones (Zou 1995), the prosperity and depression of dynasties (Zhang et al. 2004), and the frequency of disturbance events (Ye et al. 2004). Some national research also analyzes the impact of climate change on social unrest and dynastic transition in ancient China (Zhang et al. 2005; Ge 2011). All of the above research emphasizes the impact of climate change or extreme natural disasters on society. Discussions on impact dynamics and adaptation are still scarce.

In recent years, new progress has been made on this research area in China. Some scholars note that the social background, government decision-making and social response should receive more attention to further understand the impact dynamics, with the exception of the corresponding relationship between extreme climatic events and social events (the events which impact the development of society such as population fluctuation, violent conflict, replacement of dynasties etc.). Many Chinese historical case studies reflect that social responses and governmental decision-making play obvious roles in impacting the process of climate change. These studies control the level of impact, the manner of response to a certain extent, and the final outcome. These factors differ between flourishing and declining periods of society (Xiao et al. 2011, 2012; Ye et al. 2012). The influence of historical climate change on food security and social vulnerability are gradually being recognized. Fang et al. (2014) presented a new theoretical concept based on research on the impact of historical climate change on historical agricultural society.

The turn of the nineteenth century was characterized by a period of little ice ages when the climate was cooling down. For the Qing dynasty in China, the turn of the nineteenth century was also a time of transition from a period of flourishing society to a period of decline society (Fang et al. 2013). This paper analyzes the disaster relief process and the social response for two floods in China, the Yongding River flood in 1801 and the Yellow River flood in 1841 using methods of historical documents analysis and qualitative comparative analysis. These two floods reflect different response processes between the national and provincial capitals during this stage of climate cooling and social transition in the Qing dynasty. This paper explores the functions of governmental decision-making and measures in response to these flood events. Comparative studies of historical climate events and human response processes are helpful to better understand human adaptation mechanisms and to provide a reference for the modern decision making and for reducing social vulnerability.

## Methods

### Study area

**Fig. 1:**
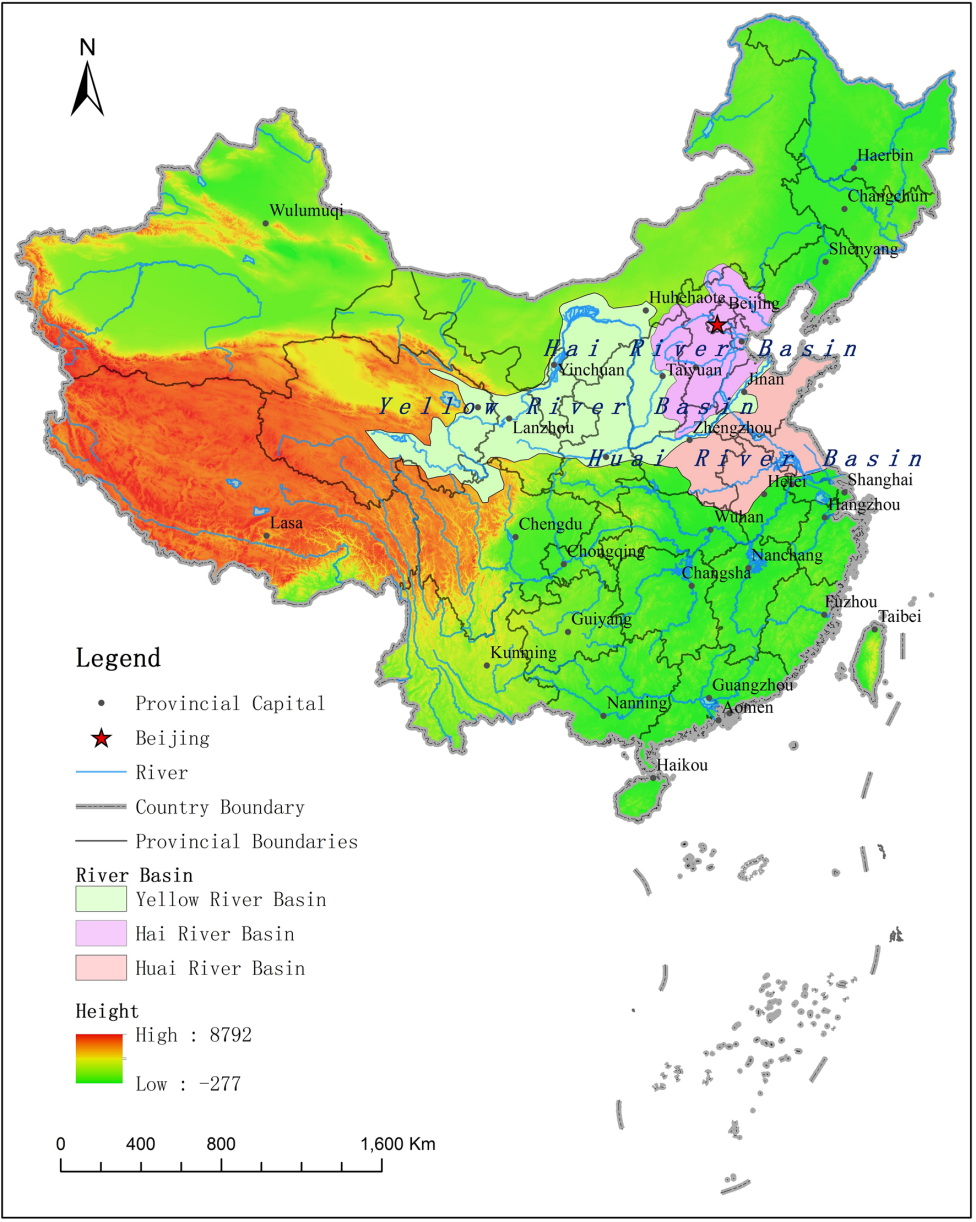
Location of study areas

The two flood events in our study occurred in the Yongding River and Yellow River basins (Fig. 1). This study focused on the comparison of impact and response processes between the national and provincial capitals, which are located along the Yongding River and the Yellow River, respectively.

The Yongding River basin lies between 39°N 112°E and 41°N 118°E. It is the largest river in the Hai River basin and flows through five provinces or autonomous regions: Shanxi, Inner Mongolia, Hebei, Beijing and Tianjin. It is one of the four main rivers that receives more attention with regard to flood prevention in China. In the Yongding River basin, the inter-annual variability of precipitation is apparent and distributed unevenly over time and space. Some areas have frequent heavy rain, which can easily cause natural disasters. Upstream of the Yong-ding River, the slope is steep, and the water is fast flowing. When this water flows downstream to the Beijing plain, the terrain is flat and the river channel so irregular that it was referred to as the Wuding River throughout history; hence, flood disasters often occurred. Since Jin dynasty (1115–1234 A.D.), government flood prevention measures included the building of dikes to avert floods and the construction of a dam to divert water. During the Kangxi Kingdom in Qing dynasty, the initial dike system was developed downstream of Lugou Bridge (Management Office of Yong-ding River in Beijing 2002). However, during large floods, the dam and dikes (levees) constructed to mitigate against flood water inundation were sometimes over topped and breached, especially in the capital city located at the middle and lower reaches of the Yongding River.

The Yellow River flows through nine provinces or autonomous regions: Qinghai, Sichuan, Gansu, Ningxia, Inner Mongolia, Shaanxi, Shanxi, Henan and Shandong. The annual sediment discharge in the Yellow River was once as high as 1.6 billion tons, and the annual sediment content is close to 40 kg/m^3^, which is the highest in the world (Ren 2006). Large amounts of sand and less water make the lower reaches of the Yellow River a perched river channel. The riverbed is higher than the level of the dike by more than 10 m (Xie 1999; Yu 2002), which increases the frequency of dike breaching when flooding occurs. According to historical documents, flood events such as breaching and channel changes have occurred in the lower Yellow River more than one thousand times over a period of more than four thousand years (Shen et al. 1935). Breaching occurred up to three times in one year during the late Ming and early Qing dynasties in the middle of the seventeenth century (Chen et al. 2012). Once the dike of the Yellow River breaches, it can cause inundation of the part of or the whole Huang-Huai-Hai plain (Fig. 1), resulting in large-scale crop failures that affect hundreds of thousands or even millions of people. The ancient capital city of Kaifeng in Henan Province is located at the apex of an alluvial fan and suffers from the most severe flood disasters. Seven floods have inundated Kaifeng city since the fourth century B.C. (Qiu 1999).

### Data sources

The information regarding natural disaster impacts, relief measures and social responses in the capital of Zhili (currently Hebei, Beijing and Tianjin) and Kaifeng come primarily from *Relief chronicles authorized by emperor in XinYou* (Qing 1802) and *Flood description in Bian Liang* (Li et al. 2006). History records in Qing dynasty Rivers and canals annal (Zhao 1927), which recorded channel changes, dike breaching, government response and treatment related to the Yellow River during the Qing dynasty.

*Relief chronicles authorized by emperor in Xin You* is a historical text about official famine policy written by individuals appointed by the Jiaqing emperor (Shao 2006); it is included in *The Chinese famine policy book (series 1, volume 2)* (Li and Xia 2003). The book consists of 38 volumes containing more than 200,000 words. It is edited in chronological order according to the Jiaqing emperor’s edicts and ministers’ reports from volume 1 to volume 38. All of the Yongding River flood situations and relief processes from July 12, 1801 to September 9, 1802 are recorded. Therefore, these records provide a clear sequence and progression of events.

*Flood description in Bian Liang* includes a specific description of the flood in Kaifeng during the summer of 1841. A daily diary totaling eight months is provided from August 2, 1841 to March 27, 1842. At the end of the book, reports and imperial edicts about this flood in *Continuous Water Jin Jian* (China Institute of Water Resources and Hydropower Research 2004) and *Actual Annals of Qing Dynasty True Records of Emperor Xuanzongcheng in Qing Dynasty* (Zhao 1986) are also attached. In addition, a time series of governmental flood relief efforts during the Qing Dynasty are sourced by Huang (2013).

### Methodology

**Fig. 2:**
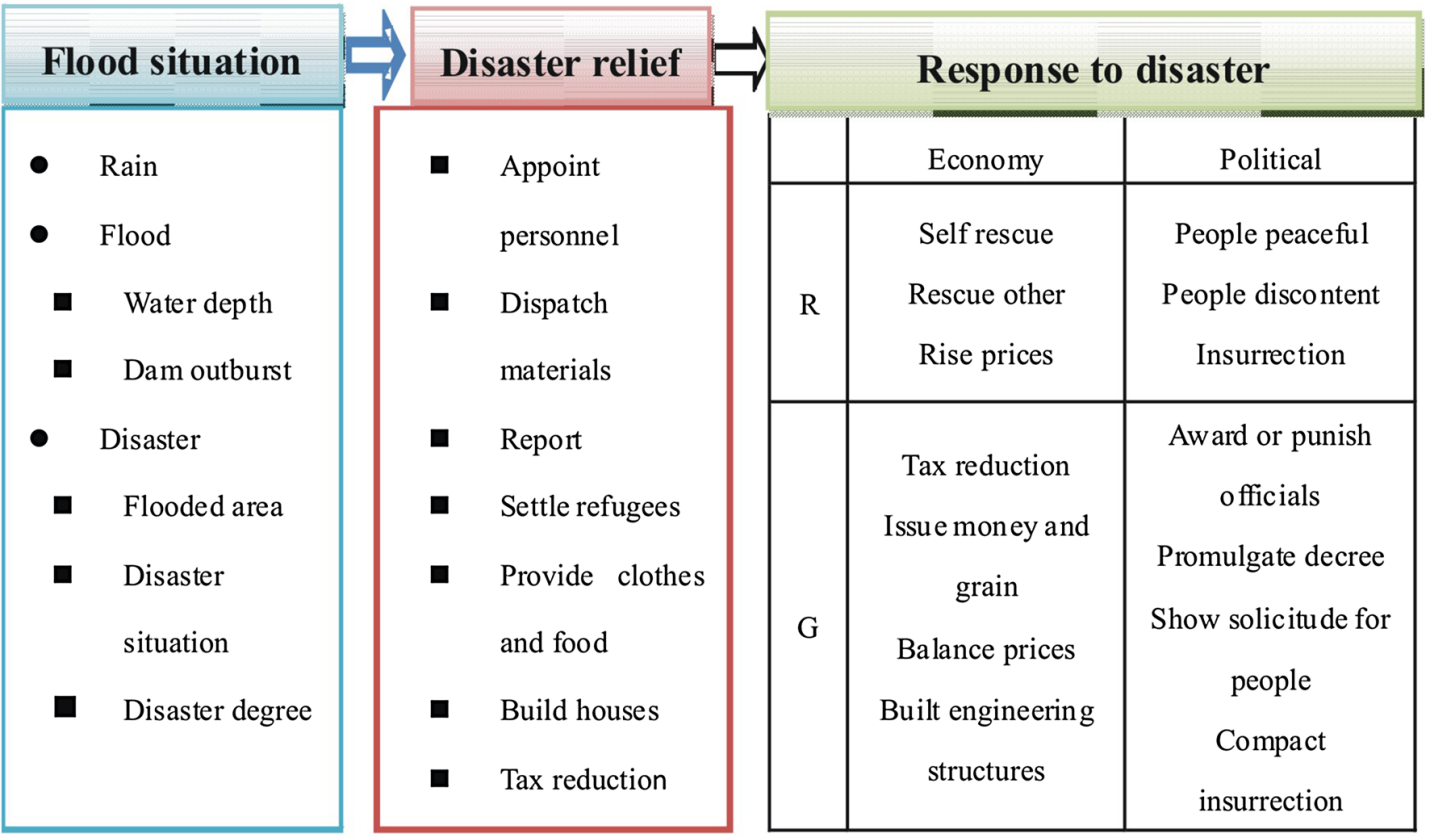
Classification of information on disaster relief and response to disaster (*R* Republic *G* Government)

**Fig. 3:**
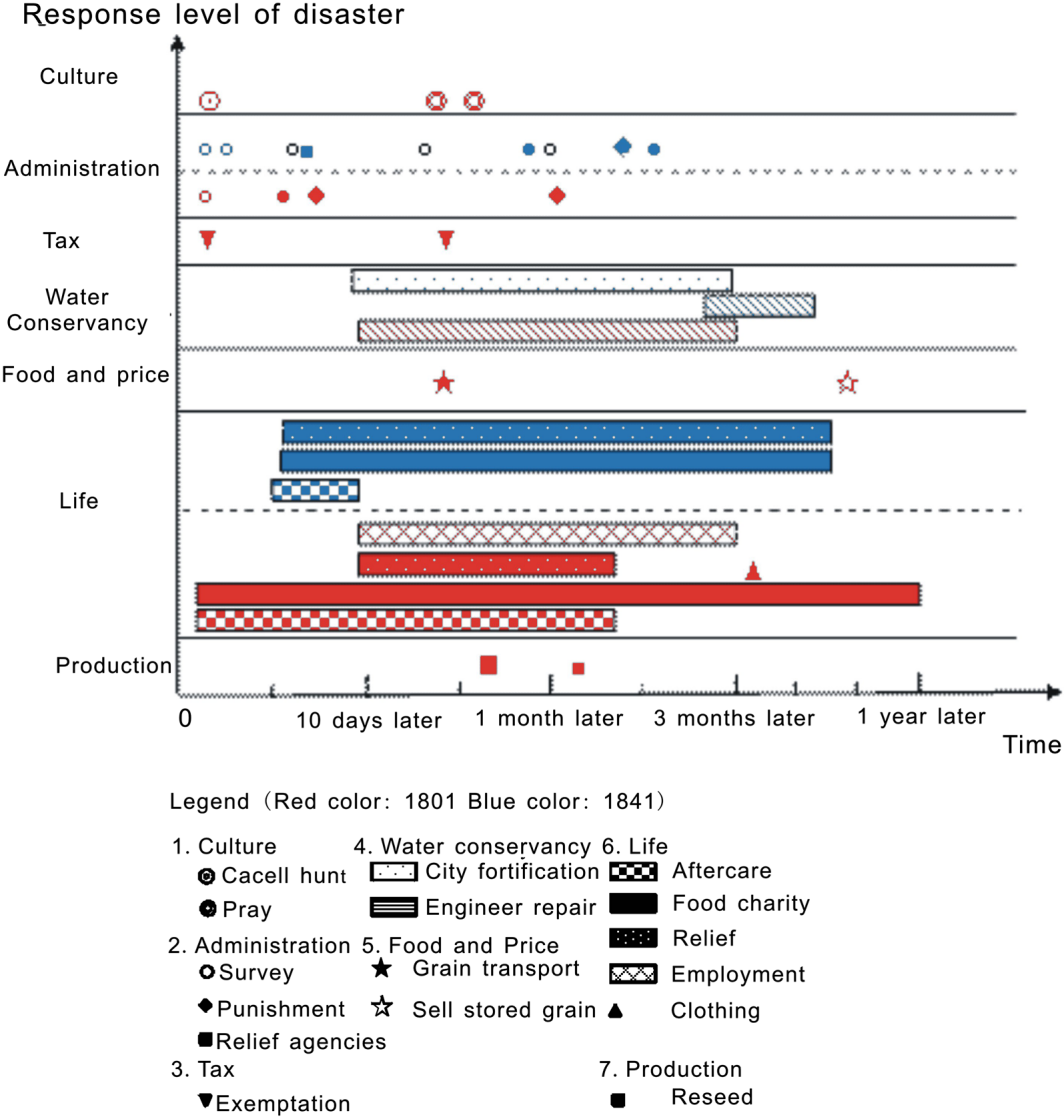
Comparison of the response and recovery measures and effects of the two floods

To analyze mechanism of social response during the two floods, the capital region Zhili (present-day Hebei Province, Beijing City and Tianjin City) and Kaifeng city in Henan province were used as examples, and detailed information on social assistance, disaster response during the flood and protection after the disaster was examined and classified using historical document analysis methods (Fig. 2). Then, a time series of recovery processes in response to these two floods was reconstructed using a the sequence of 10 days later, 1 month later, 3 month later and one year later (Fig. 3). Response and recovery processes are classified into different areas including production, life, food and prices, water conservancy, tax, administration, and culture. Finally, government response measures and disaster impacts at different stages of the flood were compared and analyzed.

## Results

### The two flooding events and the resulting hazards

Heavy summer rainfall occurring in two stages (July 10–16 and July 24–August 1) is the main cause of flooding in the Yongding River in 1801. The rainstorm in the first period led to the saturation of soil water, rising water level in the river, and river bank erosion and breach, which caused the heavy flood. After the rainstorm stopped, the flood abated but did not recede. On 25 July, heavy rain poured down again and torrents scoured the channel and road, seriously aggravating the effects of the flood disaster. After 2 August, the rain ended, and water level in the river gradually returned to normal. The flood of the Yongding River basin in 1801 was distributed predominantly along the Hai River, South Canal, Ziya River, Daqing River, Yongding River, North Canal, JiYun River and Luan River, affecting 160 counties in Shanxi, Inner Mongolia, Hebei, Beijing and Tianjin. Among them, Shijingshan, Wanping, Daxing, Changxindian, and Liangxiang, which were near the river’s breaching point and along the place where the flood flowed, were the hardest-hit areas (Ye et al. 2014).

The rising flow of the upper reaches of the Yellow River between July 23rd and 31st and the strong rainfall that followed from August 3rd to 22nd contributed to Kaifeng, Henan Province being surrounded by floodwaters of 1841 for 68 days in 1841. The most serious flood was in June of the lunar calendar, from 23 July to 1 August. Many states and counties along the Yellow River were seriously affected. In the records, the flood disaster in Kaifeng began with the water rushing into Kaifeng City on 2 August 1841, and ended with the closing of the breached dam on 19 March 1842. The flood of the Yellow River basin in 1841 affected almost 108 counties in Shanxi, Shaanxi, Inner Mongolia, Ningxia, Shandong, Henan and Anhui. The most serious impacts occurred in lunar June, from July 23rd to August 1st. The River in Zhangjiawan breached, and Kaifeng city was surrounded by the flood. In late lunar June, the lower reaches of the Yellow River were cutoff, and the flood then flowed into the Huai River, causing breaching. This flood impacted 61 counties in Shanxi, Shaanxi, Henan, Anhui and other provinces.

### The relief and response speed to the two foods

Flood relief occurred differently for each event. In 1801, flood relief was led by the government, especially the central government. In 1841, local gentries played an important role in the early stages of food relief, from the first rescue to the management of the relief factory. The rescue work in the late period was taken over by engineer soldiers. At the same time, local officials served as relief directors throughout the flood event.

For example, in terms of surveying and reporting, the emperor of Jiaqing sent ministers to the disaster area from the west, east, north and south roads on the 7th day (July 17th) after the flood in 1801. The ministers had to guide local officials to carefully survey the flood and produce detailed and accurate reports. In addition to the breaching of the Yongding River, the extent of damage to cropland was assessed and reported with villages as the basic unit. In 1841, disaster surveying and reporting was complex. Central and local government officers and local gentries, especially local officers, surveyed the disaster situations. The Daoguang emperor instructed Wen Chong, the director of the east river channel, Niu Jian, the inspector of Henan province and the scholar Wang Ding in succession to survey the disaster situations.

The speed of the response varied. In 1801, the emperor ordered a thorough survey after 7 days; in 1841, the emperor did not send officials to supervise for 18 days, and officials arrived in Kaifeng after 40 days. In 1801, central officials were divided into four groups and ordered to assess and survey the disaster area 7 days after the flood. In 1841, however, complete surveying began very late, and the disaster situation was not reported until three months later.

### The impact and mechanisms of the two floods

First, agricultural production was a key process in the flood response chain in both Zhili and Kaifeng, and the two floods both influenced society by impacting agriculture, food, materials and other aspects. Second, the level of the impact and subsequent related processes of the two floods were relatively consistent. A series of consequences generated by the floods ranged from reduced agricultural production and damage to water conservancy establishments to rising prices, vagrants and refugees, and robbery. Finally, the two floods both affected social stability to some extent, especially without timely national assistance, although there were also some differences in the social impact between the two floods.

### Response and recovery measures

Response and recovery measures were different in the two floods due to differences in the objects affected (Fig. 3). In 1801, the flood affected farmland and created many refugees. Governmental response actions involved in all aspects of society, including production, housing, food prices, tax, and water conservancy and administration, and even the emperor’s personal behavior and culture. The government successfully organized a series of relief measures, including a set of rice porridge factories, tax exemptions, crop reseeding, engineer employment provisions, and relief substitution. National food relief during the flood season and extending into the following year effectively guaranteed the lives and social stability of the people. In 1841, the government made it a priority to protect Kaifeng city from the flood. The response measures were relatively simple, focusing mainly on providing shelter and food for victims. During the flood, food prices increased and robberies occurred even in the daytime; however, the government could do nothing but issue announcements to address it.

### Flood mitigation infrastructure

The government organized the repair of water utilities after the floods. In 1841, the work mainly focused on flood emergency rescue, making urgent repair on the dam around Kaifeng city; however, in 1801, the government carried out more comprehensive disaster prevention measures such as channel dredging. In the flood of 1801, after completing repair work, the government employed the poor in digging the Tonghui River, repairing the Yongding River, South Canal, North Canal, and Hun River and dredging channels to prevent and mitigate impact of floods in the future.

### Differences in administrative regimes between the two floods

Differences in the impact and response processes to the two floods reflected different administrations. In 1801, the central and local governments were unified, and there was strict and impartial award and punishment principle. Moreover, the government investigated and punished corrupt officials reported by victims on many occasions.

In 1841, problems delayed the construction of the dike outside the city. For example, officials blamed each other because of differing views on flood management, officers became corrupted and cut corners during the dike construction, engineer soldiers and gentries did not like working with each other and soldiers fought with citizens; all of these problems hampered the progress of the relief work.

All of above, although it is still unclear if the 1801 Yongding River Flood was of equivalent magnitude hydrologically and in some quantitative terms of societal impact to that of the Yellow River of 1841, we assume they were all heavy hydrological floods resulted by rainstorm and extensive region was seriously affected according to the abundant documental descriptions on hydrologic process and affected disaster areas. By the above comparison of these two floods, it confirmed the central conclusion of this study, ineffective government response to the flood disaster resulted in substantially more adverse impacts to the affected populations.

## Discussions

The climatic background of the two floods is essentially the same. The climate began to cool during the nineteenth century, and the frequency of flood and drought disasters increased. Climate instability increases the burden of social disaster response. There was a clear contrast, however, between the two floods in terms of magnitude of the flood, the disaster relief and the degree of attention drawn at a national level due to differences in regional and political status.

### The role of climate variability

The two floods occurred in the first half of the nineteenth century, when the climate was cold. The average winter temperature was more than 1 °C lower than the present temperature in eastern China (Ge et al. 2002). During this period, there were also significant changes in precipitation. There were more flood and drought disasters in the first half of the nineteenth century than that in the eighteenth century.

According to the records of precipitation and flood disaster processes, the likely cause of both floods was the same—continuous heavy rainfall in the summer. The disaster processes, however, were different. In 1801, heavy rainfall was the determinant of the flood disaster. The Yongding River rose from the beginning of the heavy precipitation until the rain ended, and the flood gradually receded approximately one month later. In 1841, however, Kaifeng suffered for approximately 10 days after the flood in the lower reaches of the Yellow River. Heavy rainfall in Shanxi and Shaanxi led to rising water in the upper reaches of the Yellow River and its tributaries, which was the main cause of the flooding in Kaifeng, and consequently Kaifeng was surrounded by the flood for more than one month after the heavy rain ended.

Overall, the climate began to cool down during the nineteenth century, and the frequency of flood and drought disasters rose. Continuous heavy rainfall in the summer frequently leads to river rose and flood waters surrounding the cities along the river. All climate instability increases the burden of social disaster response.

### National strength reflected by the strength of governmental food relief

The flood in 1801 occurred during the transition from the “Kangxi-Qianlong Kingdom Flourishing Age” (1681–1796) to the “Jiaqing-Daoguang Kingdom Decline Age”, when the territory and economic strength was at its peak in the Qing dynasty on the verge of decline. The flood in 1841 occurred on the eve of the Opium War. Financial shortages, official corruption, army laxity and domestic and foreign crises brought about the rapid decline of this prosperous feudal state.

The strength of governmental food relief for these disasters is also a reflection of national strength. The quantity of national food relief refers to total weight of governmental food transfer which unit is dan (1 dan equals 28 kg in the Qing Dynasty). National food relief strength is one of indexes of showing disaster relief effort, which is calculated by quantity of national food relief divided by the disaster index. In the Qing dynasty, the quantity and strength of governmental food transfer in the north China plain was highest from 1740 to 1799; the annual average national food relief was 356,000 dans, and the average governmental food transfer strength index was 17.66. From 1800 to 1869, however, food relief was declining, and the annual average governmental food transfer was only 70,400 dans with an average national food relief strength index of 2.91. The Qing government spent 1135,000 dans of grains on drought relief in 1785, but the amount of food relief decreased in the 1790s, with an annual average value of 287,400 dans. The amount dropped dramatically in the early 1800s, reaching minimum values of 18,800 and 10,000 dans in the 1830 and 1840s, respectively. During the 1830–1840s, the government did not organize powerful, effective food relief for the most severe floods and droughts, with the exception of the floods in 1822 and 1823, when the government offered 1161,000 dans of relief grains (Huang 2013). In conclusion, the national government in 1801 was stronger than in 1841; the Qing government had maintained a more comprehensive disaster response effort in 1801.

### Different political statuses reflected by disaster response and recovery measures

The differing political statuses of disaster areas also led to differences in disaster response and recovery measures. As the political center of the Qing dynasty, Zhili (including present-day Hebei Province, Beijing City and Tianjin City) was a particular priority of the government and received the greatest and fastest food relief. The amount of food obtained by Zhili accounted for more than 50% of the total amount in the north China plain of the Qing dynasty. As the second-level administrative center, Henan province received less attention and food, although the northern zone served as a food distribution area in Henan. In 1801, tax exemption and food relief were still important disaster relief strategies. States and counties in the capital area enjoyed tax concessions according to the degree of damage. The total amount of flood relief food was 900,000 dans, mainly from Caoliang (rice transported to the capital by water). A small amount of food was bought from eastern Henan and northeastern China (Huang 2013). The government paid over 1,000,000 liang (1 liang equals 50 g) of silver for the Yongding River riverbank engineering in this year, which was the largest relief effort of the Qing Dynasty (Wang 2007). The scope and strength of the national relief in 1841 were much lower than those in 1801.

Generally speaking, the two floods had different national strength backgrounds. Different national disaster relief efforts were enacted to varying degrees, and the floods received different levels of national attention due to differences in regional and political status. These are all possible impact factors causing different responses during the two floods.

### Significance of this research for contemporary flood hazard

Various flood relief measures and disaster management regimes can be used as a reference for responding to flood events. First, when flooding occurs, the government can organize a series of relief measures, including providing food and clothes, tax exemptions, crop reseeding, engineer employment provisions, and relief substitutions. Second, the impact conveying process and influence degree of the floods were relatively consistent. Agricultural production was a key process in the flood response chain, and the two floods both influenced society by affecting agriculture, food, materials and other aspects. Thus, contemporary flood hazard mitigation should follow the order of flood response chain, from measures on production, food, economy to society. All levels of relief measures should be implemented, including food relief, agriculture production, housing, food prices, tax, water conservancy and administration. Especially, governmental food relief and support of money, seed or technology for agriculture production in modern flood hazard mitigation are very necessary. Third, central and local governments should be unified and cooperate with each other, and officers should be rewarded or punished according to their performance in coping with flood hazard. The strict principle of reward and punishment should be implemented as a policy by legislation.

## Conclusions

This study compared two floods that both occurred during the transition from warm to cold climate patterns during the Little Ice Age. The disaster responses processes were compared. It confirmed: the disaster relief systems had a significant effect on the consequences of the floods. The response and recovery processes of the Zhili flood in 1801 and the Kaifeng flood in 1841 were different.

1. The central government assumed a lead position, from flood surveying to relief processes, in 1801; local officers were coordinated and guided by the central government. In 1841, local government played an important role, and local gentries also contributed to surveying and relief under the supervision of the central government.
2. The response and recovery measures in 1801 were diverse and effective and operated on many levels; the strategies included forming rice porridge factories, creating tax exemptions, reseeding croplands and providing employment to the public. In 1841, the government prioritized preventing Kaifeng city from being surrounded by flooding. The government focused on providing shelter and food for victims.
3. National food assistance during the flood season in 1801 and even in the following year effectively guaranteed peoples’ lives and social stability. Unfortunately, serious public security hazards such as daytime robberies still appeared in 1841.
4. From the aspect of water conservancy, the government carried out inspections and comprehensive disaster prevention measures such as dredging channels after the flood in 1801. In 1841, however, the work mainly focused on flood emergency rescue.
5. The flood in 1801 was controlled by a central authority. Materials transportation, disaster monitoring and punishment work were well executed during the flood. The authorities effectively controlled refugees and sustained a relatively stable regime. In 1841, the officials were corrupt and officers in different ranks blamed each other.

The climatic background of the two floods is essentially the same. However, there was a clear contrast between the two floods regarding the background of national strength, the stage of national disaster relief and degree of national attention due to differences in regional and political status. The flood in 1801 occurred when the prosperity of the Kangxi-Qianlong kingdom had just ended, and 20 years after the turning point of climate cooling. The flood in 1841 occurred when society had declined and was approximately 30 years from flourishing. These are all possible impact factors in the different responses during the two floods.

## Abbreviation

IPCC: International Panel of Climate Change
